# The value of MR imaging in posttraumatic diffuse axonal injury

**DOI:** 10.4103/0974-2700.42204

**Published:** 2008

**Authors:** Luigi Beretta, Marco Gemma, Nicoletta Anzalone

**Affiliations:** Department of Head and Neck Anesthesia and Intensive Care for Head and Neck Surgery Hospital S. Raffaele, Milan, Italy; 1 Neuroradiology Hospital S. Raffaele, Milan, Italy

A 40-year-old man was involved in a road accident. He was comatose from the outset (GOS = 4) and was intubated and ventilated upon admittance to our hospital.

**Figure 1:**
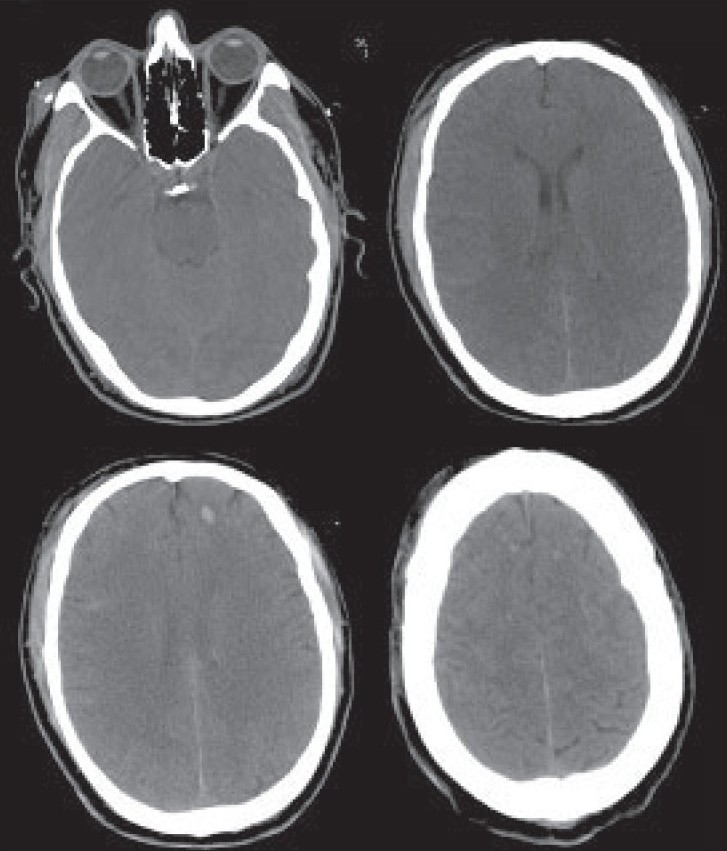
Brain CT scan showing few small cortical contusions. There are no mass lesions or midline shift; the ventricles and perimesencephalic cisterns are normal

A brain CT scan showed only few small cortical contusions; neither mass lesions nor midline shift were apparent; the ventricles and perimesencephalic cisterns were normal [Figure 1]. CT scan is considered the first-choice diagnostic procedure in head injury patients. Nevertheless, it cannot account for the severity of coma in some cases.

**Figure 2:**
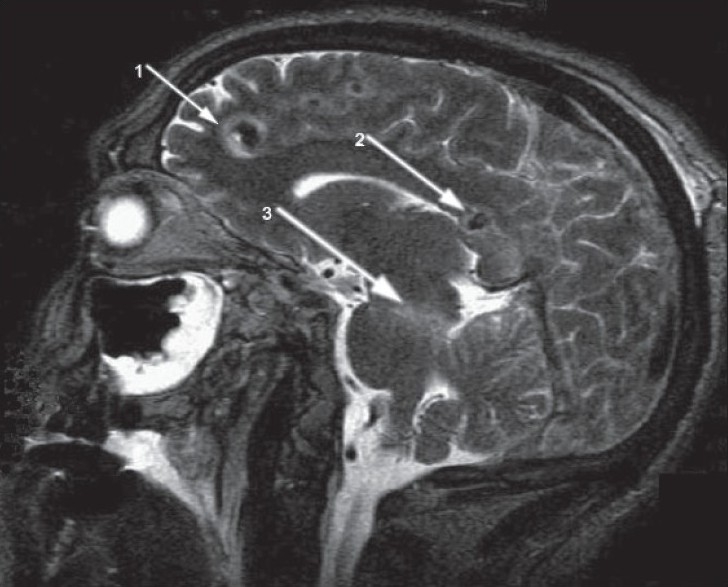
MR T2 sagittal image showing multiple cortico-subcortical frontal hemorrhagic contusions (arrow 1); a hemorrhagic contusion in the posterior part of the corpus callosum (arrow 2); and edema of the splenium and contusion of the dorsolateral part of the brainstem (arrow 3)

MR examination was performed on the third day after trauma. A T2 sagittal image [Figure 2] showed multiple cortico-subcortical frontal hemorrhagic contusions (arrow 1) together with a hemorrhagic contusion in the posterior part of the corpus callosum (arrow 2); this was associated with edema of the splenium and contusion of the dorsolateral part of the brainstem (arrow 3). These findings suggested severe diffuse axonal injury (DAI).

DAI is a type of brain damage which is secondary to rotational acceleration / deceleration.[1] Centripetal forces, usually generated by high-speed impact, lead to cortico-subcortical shearing. As the severity of such forces increases, deeper areas of the brain are progressively involved, such as the corpus callosum and the brainstem. The resultant damage is axonal since the axons are torn or stretched such that axoplasmic transport and the electrical network become impaired.

Animal studies, neuropathology, and neuroradiology have classified DAI into three stages.[2] The involvement of the grey-white matter junction indicates stage I, corpus callosum involvement indicates stage II, and brainstem involvement indicates stage III. Clinical studies demonstrate that the prognosis becomes poorer as deeper structures are involved.

MR can identify DAI lesions, while CT scan often fails to do so. MRI provides clues to prognosis and to the physiopathology of the ensuing coma. In fact, CT scan can be completely negative in patients with DAI and no subtle CT findings in DAI have yet been described. Moreover, even repeated CT scans are of no value in suggesting DAI at any time after trauma.

**Figure 3:**
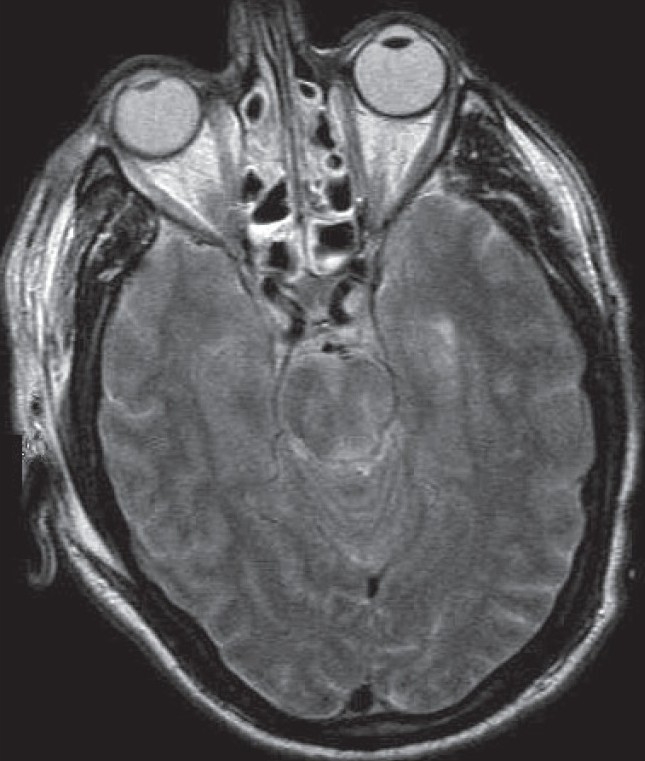
MR T2 axial image showing contusion of the brainstem

The involvement of the anatomical structures characterizing each of the three stages is usually visible in distinct slices of MR imaging, with brain stem lesions being seen in the axial view [Figure 3].

In our patient, it was possible to diagnose DAI III through a single MR sagittal slice, in which all three injury levels—parasagittal subcortical lesions and corpus callosum and brainstem lesions—were visible.

The outcome of the patient 6 months after trauma was scored as ‘severe disability’ on the Glasgow Outcome Scale (GCS).

It is well known that DAI is an MR diagnosis, and MR imaging is recommended in posttraumatic coma unexplained by CT scan, both for diagnosis and for assessing prognosis. Since treatment in the acute posttraumatic phase is not affected by the MR diagnosis of DAI, it is advisable to perform MR after sufficient hemodynamic and respiratory stability has been achieved. It should be noted that, while diffusion-weighted MR imaging may be diagnostic even in the first few hours after trauma, MR T2 imaging allows good assessment of DAI from about 12 h after trauma. Our patient was unusual in that a single MR T2 sagittal slice showed all of the three types of lesions that characterize stage III DAI.
